# Tumor-Associated Macrophages and Regulatory T Cells Infiltration and the Clinical Outcome in Colorectal Cancer

**DOI:** 10.1007/s00005-017-0463-9

**Published:** 2017-03-25

**Authors:** Dariusz Waniczek, Zbigniew Lorenc, Mirosław Śnietura, Mariusz Wesecki, Agnieszka Kopec, Małgorzata Muc-Wierzgoń

**Affiliations:** 1 SHS in Katowice, Department of Propaedeutics Surgery, Chair of General, Colorectal and Polytrauma Surgery, Medical University of Silesia in Katowice, Żeromskiego 7, 41-902 Bytom, Poland; 2 SHS in Katowice, Chair of General, Colorectal and Polytrauma Surgery, Medical University of Silesia in Katowice, Plac Medyków 1, 41-200 Sosnowiec, Poland; 3 Tumor Pathology Department, Maria Sklodowska-Curie Memorial Cancer Center and Institute of Oncology, Gliwice Branch, Armii Krajowej 15, 44-100 Gliwice, Poland; 4 Department of Internal Medicine, Medical University of Silesia in Katowice, Żeromskiego 7, 41-902 Bytom, Poland

**Keywords:** Tumor-associated macrophages, TAMs, Tregs, Regulatory lymphocytes, Colorectal cancer

## Abstract

The aim of the study is the assessment of the intensity of the infiltration of tumor-associated macrophages (TAMs) CD68^+^/iNOS^−^ and Tregs CD8^+^/FoxP3^+^ in colorectal cancer (CRC) patients as prognostic factors with respect to disease-free survival (DFS) and overall survival (OS). In this retrospective study, tissue samples were obtained from 89 patients undergoing resection for CRC (stage IIA, pT3N0M0 and stages IIIB and IIIC, pT3N1-2M0). Recurrence was observed in 45 patients at the time of the follow-up (10 local recurrences, 35 distant metastases). In patients with recurrence the following were present: a tendency to an older average age at the time of diagnosis (*p* = 0.07), higher nodal involvement (*p* = 0.002) and more advanced clinical disease (*p* = 0.01). The analysis of the clinical data and immunohistochemical studies were performed with the methodology of identification of TAM and Treg subsets in histological sections, with the aim to use it in routine clinical management. Both DSF and OS were the clinical parameters assessed in the study. The presence of intense infiltration of TAMs in the tumor stroma was related to shorter DFS (*p* = 0.005) and OS (*p* = 0.006). The opposite tendency was observed in the tumor front (*p* = 0.061). The relative risks of recurrence and cancer-related death were more than twice higher in the group of patients with intense infiltration of TAMs in the tumor stroma (RR 2.05, 95% CI 1.33–3.14; *p* = 0.001 and RR 2.08, 95% CI 1.28–3.39; *p* = 0.003, respectively). Intense infiltration of Tregs in the tumor stroma was related to shorter DFS and OS (*p* < 0.0001). The relative risks of recurrence and death in a group of patients with intense infiltration of Tregs in the tumor stroma were more than 12 times higher than in patients with less intense infiltration (RR 12.3, 95% CI 5.44–27.9; *p* < 0.0001 and RR 12.5, 95% CI 4.9–32.4; *p* < 0.0001, respectively). Infiltration of TAMs CD68^+^/iNOS^−^ and Tregs CD8^+^/FoxP3^+^ in the tumor stroma are negative prognostic factors with a positive correlation between them. Tregs may constitute an independent prognostic factor in patients with CRC.

## Introduction

Colorectal cancer (CRC) is one of the most common types of cancer and it constitutes one of the main causes of death. This form of cancer is the third most common cancer in the world, with nearly 1.4 million new cases diagnosed in 2012 (Ferlay J et al. 2014). The pathogenesis of CRC in the aspect of the tumor microenvironment is understood as a complex model of mutual relationships and a complex of interactions between developing cancer and the surrounding tissue, including the immune system (Muc-Wierzgoń et al. 2014; Piktel et al. 2016). At present, it is confirmed that the type, the location and the density of immune cells infiltrating the tumor are the manifestations of the immune response within the tumor environment. Both mechanisms of innate and acquired immunity play a significant role in tumor development and progression. Consequently, they influence the course of disease (Ohtani 2007; Pages et al. 2005). Tumor-associated macrophages (TAMs) are a component of the innate immunity, whereas subpopulations of regulatory T-lymphocytes (Tregs) are a part of the acquired immunity. Changes in the number of subpopulations of TAMs and Tregs and various relationships between them are observed in CRC patients. It seems that within the tumor microenvironment TAMs polarize toward tumor-promoting phenotypes. By activation of direct and indirect mechanisms due to the inability to present antigen, TAMs predominantly represented in the tumor by the M2 phenotype induce the creation of lymphocytes showing the expression of the forkhead box P3 transcription factor (FoxP3^+^). These lymphocytes are responsible for immune tolerance of the system to the tumor (Gabrilovich et al. 2012; Galon et al. 2006; Ramanathan et al. 2008; Shih et al. 2006). This mechanism may create favorable conditions for tumor development and it promotes further neoplastic progression (Zou 2006). However, in the case of CRC, which is largely inflammation-dependent tumor, these relationships are considerably more complex and more difficult to predict. The authors made an attempt to use the quantitative and phenotypic assessments of the previously mentioned components of the immune system as additional prognostic factors in patients in whom commonly used prognostic factors fail for a number of reasons.

The aim of the study was the assessment of the intensity of M2 macrophage infiltration defined as CD68^+^/iNOS^−^ and the intensity of CD8^+^/FoxP3^+^ lymphocyte infiltration as prognostic factors for disease-free survival (DFS) and overall survival (OS) in patients who underwent surgical treatment due to CRC.

## Materials and Methods

### Study Groups

Eighty-nine patients who were primarily operated on due to CRC between 2008 and 2011–stage IIA (pT3N0M0) and stages IIIB and IIIC (pT3N1-2M0) were enrolled in this retrospective study. The enrollment was performed in a manner providing a similar number of patients with confirmed recurrence and without symptoms of recurrence during the follow-up. The exclusion criteria were as follows: patients >80 years of age, young patients in whom genetic background of the disease had been confirmed (familial adenomatous polyposis and hereditary nonpolyposis CRC), patients treated due to autoimmune and rheumatoid diseases. Patients with oncological history due to another type of tumor were also excluded from the study.

The clinical parameters analysed in the present study included DFS which was defined as the time from surgery to the occurrence of local relapse or distant metastases and OS. The results of immunohistochemical staining as well as complete clinical data on disease progression and the postoperative course were obtained for all of the analyzed cases. Recurrence at the follow-up was observed in 45 patients. Ten of 45 patients presented with local recurrence or recurrence in the abdominal wall. In the remaining 35 patients, distant metastases were observed, mainly to the liver (29 cases) and to the lungs (6 cases). A tendency to an older average age at the time of diagnosis (*p* = 0.07) as well as higher nodal involvement (*p* = 0.002) and higher clinical progression of the disease (*p* = 0.01) was observed in patients with recurrence. The complete characteristics of the study groups are presented in Table 1.

**Table 1:** Clinico-pathological characterization of CRC patients enrolled in the study in groups depending on evidence of the recurrence

Feature	Patients with recurrence, *n* = 45	Patients with no recurrence, *n* = 44	Statistical significance
Median and age range (years)	65 (40–84)	69 (45–85)	*p* = 0.07
Gender	F: 22 (48.9%) M: 23 (51.1%)	F: 22 (50.0%) M: 22 (50.0%)	*p* = 0.92
Tumor location	Right intestine: 11 (24.4%) Left intestine: 34 (75.6%) Including: caecum: 9 (20.0%) ascending colon: 1 (2.2%) hepatic flexure: 1 (2.2%) transverse colon: 0 (0.0%) splenic flexure: 1 (2.2%) descending colon: 2 (4.4%) sigmoid colon: 9 (20.0%) sigmoid colon-rectum: 3 (6.7%) rectum: 19 (42.2%)	Right intestine: 19 (43.2%) Left intestine: 25 (56.8%) Including: caecum: 8 (18.2%) ascending colon: 7 (15.9%) hepatic flexure: 3 (6.8%) transverse colon: 1 (2.3%) splenic flexure: 1 (2.3%) descending colon: 4 (9.1%) sigmoid colon: 11 (25.0%) sigmoid colon-rectum: 2 (4.5%) rectum: 7 (15.9%)	*p* = 0.13
Clinical stage at the time of surgery (TNM classification)	T1: 0 (0.0%); T2: 2(4.4%); T3: 41 (93.2%); T4: 2(4.4%) M0: 45 (100%) N0: 15 (33.3%) N1: 17 (37.8%) N2: 13 (28.9%)	T1: 0 (0.0%); T2: 0 (0.0%) T3: 44 (100.0%); T4: 0 (0.0%) M0: 44 (100%) N0: 28 (63.6%), N1: 14 (31.8%), N2: 2 (4.5%)	*p = 0.002 for N feature
Clinical stage	II: 15 (33.3%) III: 30 (66.7%)	II: 28 (63.6%) III: 16 (36.4%)	*p = 0.01
Adjuvant treatment	Treated: 26 (47.8%) Not treated: 19 (42.2%)	Treated: 24 (54.6%) Not treated: 20 (45.4%)	*p* = 0.17
Histological malignancy grading	G1: 1 (2.2%). G2: 34 (75.6%). G3: 10 (22.2%)	G1: 3 (6.8%). G2: 33 (75.0%). G3: 8 (18.2%)	*p* = 0.54

*Statistically significant differences between groups were observed in the N feature of the TNM classification and clinical staging

### Immunohistochemical Staining

In order to visualize macrophage infiltration within the tumor and at the tumor front, we used the CD68 marker showing the expression in all macrophage-like cell types regardless of their phenotype (both in M1 subtypes and M2 family). A number of phenotypes were described within M2 macrophages. These phenotypes differed in terms of the expression of specific markers. To visualize them, a simultaneous use of at least several antibodies would be necessary. Therefore the authors initially decided to use immunohistochemical staining of the M1 macrophages with anti-nitric oxide synthase antibody present solely in M1 macrophages in order to be subtracted from the overall number of all macrophages CD68^+^ with the intention to simplify the method without influencing its specificity. However, during the investigation the authors found only very scant infiltration of iNOS positive cells in comparison to relatively abundant CD68-positive infiltrates (<1% iNOS^+^ cells in the pool of CD68^+^ cells). Therefore, it was decided to treat the total CD68-positive pool as M2 phenotype macrophages CD68^+^/iNOS^−^ without introducing significant bias. The reader of the article should bear in mind this assumption in every case where the authors refer to CD68^+^/iNOS^−^ phenotype.

The transcription factor FoxP3^+^, which is considered a specific marker of Treg lymphocytes, was detected in the overall number of lymphocytes showing the expression of CD8^+^ surface antigen in order to determine the intensity of infiltration of Treg lymphocytes defined in this study as demonstrating coexpression of CD8^+^/FoxP3^+^ proteins.

Tissue samples collected in the form of paraffin blocks were used for immunohistochemical studies. Four immunohistochemical stainings were performed from each block containing tumor tissues, using the following antibodies: monoclonal mouse antibody anti-CD68, clone PG-M1 (catalogue no.: M 0876; Dako, Denmark), rabbit monoclonal anti-FoxP3 antibody—clone SP97 (catalogue no.: 506-3974; Zytomed Systems, Berlin), mouse monoclonal anti-CD8 antibody—clone C8/144B (catalogue no.: IR623; Dako, Denmark), rabbit polyclonal anti-iNOS antibody (catalogue no.: NB300-605; Novus Biologicals, UK). All paraffin-embedded blocks with tumor tissues were cut into 4-μm sections. Deparaffinization of the samples and tissue antigen retrieval were performed using PT-Link (catalogue no.: PT101; Dako, Denmark) and buffers of various pH: EnVision Flex Target Retrieval Solution, High pH (catalogue no.: K8024; Dako, Denmark) for specimens incubated with monoclonal anti-FoxP3, anti-CD8 and anti-CD68 antibodies and EnVision Flex Target Retrieval Solution, Low pH (catalogue no.: K8005; Dako, Denmark) for specimens stained using polyclonal anti-iNOS antibody. To avoid false-positive immunohistochemical reaction, endogenous peroxidase activity was blocked with 3% hydrogen peroxide for 5 min.

**Fig. 1:**
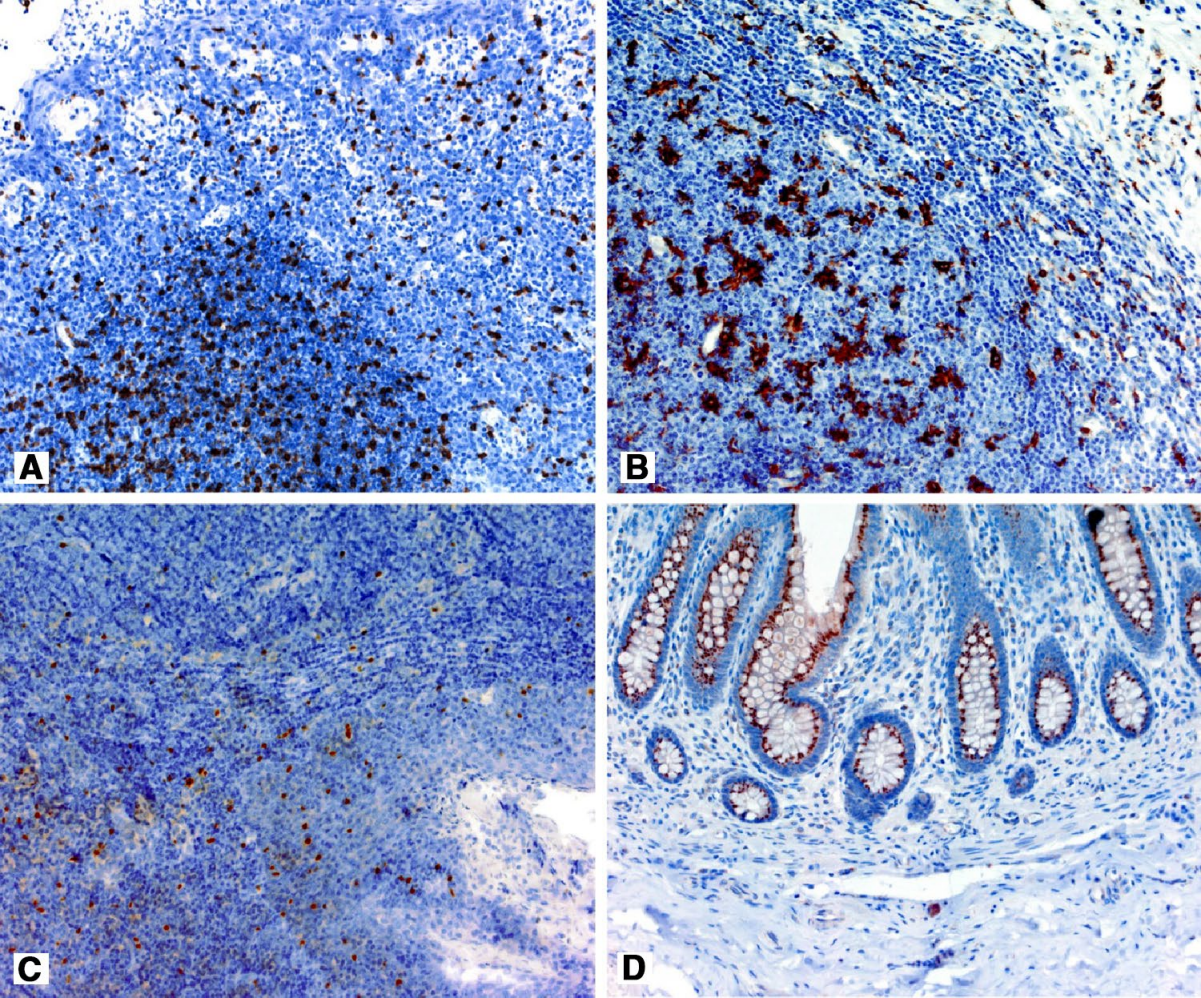
The results of CD8 positive control—tonsil (**a**), CD68—tonsil (**b**), FoxP3—tonsil (**c**), iNOS—appendix (**d**)

The technical details related to primary antibody dilutions, dilution buffer, incubation time and the type of positive control are presented in Table 2, whereas the results of staining for positive controls are shown in the Fig. 1.

**Table 2:** The summary of the most significant technical details related to immunohistochemical staining

Type of antibody	Type/clone	Diluent type	Primary antibody dilution	Incubation time (min)	Positive control
iNOS Novus Biologicals, UK	Polyclonal	Antibody Diluent with Background Reducing Components Dako, Denmark	1:300	30	Appendix
FoxP3 Zytomed Systems, Germany	Monoclonal SP97	EnVisionTMFLEX Antibody Diluent Dako, Denmark	1:150	30	Tonsil
CD8 Dako, Denmark	Monoclonal C8/144B	EnVisionTMFLEX Antibody Diluent Dako, Denmark	1:1	20	Tonsil
CD68 Dako, Denmark	Monoclonal PG-M1	EnVisionTMFLEX Antibody Diluent Dako, Denmark	1:100	20	Tonsil

EnVisionTM FLEX/HRP system was used to visualize the antigen-primary antibody reaction (catalogue no.: K8024; Dako, Denmark), consisting of a mixture of goat anti-mouse and anti-rabbit antibodies linked to polymer containing immobilized horseradish peroxidase and chromogen 3,3′-diaminobenzidine (EnVisionTM FLEX DAB + Chromogen, catalogue no.: K8024; Dako, Denmark).

The sections were counterstained using aqueous solution of hematoxylin (Mayer’s solution), they were dehydrated and closed using a permanent mounting medium (Dako Toluene-Free Mounting Medium, catalogue no.: CS705; Dako, Denmark).

### Evaluation of Immunohistochemical Reactions

The evaluation of immunohistochemical reactions was performed using a light microscope, Olympus (Tokyo, Japan) type CX41–5× and 10× magnification objectives for features rated using a semi-quantitative scale and 40× for quantitative features. The intensity of macrophage infiltration CD68^+^ was determined semi-quantitatively using a 4-point scale based on the following criteria: 0—negative, 1—weak, 2—moderate, 3—intense. The intensity of infiltration was assessed in the areas with the highest presence of infiltration (hotspots), separately within the tumor and in the infiltrating front of the tumor.

Due to the low number of positive cells, the presence of iNOS^+^ was determined under 40× magnification in the areas corresponding to the most intensified infiltration of CD68^+^.

In the case of CD8^+^ lymphocytes, their absolute number in the tumor stroma was counted within one high power field (hpf) under 40× magnification (hpf diameter of the optical track of the microscope was 22 mm). Only the fields with the highest intensity of infiltration within the tumor stroma were selected for the assessment. A similar methodology was applied to FoxP3^+^ lymphocytes, choosing the areas in the consecutive serial paraffin-embedded section that analogically corresponded to the areas selected at the time of CD8^+^ lymphocyte counting. In rare cases when the number of FoxP3^+^ lymphocytes was higher than the number of CD8^+^ cells, the higher value was adopted for both parameters. The adopted methodology constitutes a simplified method of the assessment of the intensity of CD8^+^/FoxP3^+^ infiltration based on the phenotype averaged for the entire area and it definitely does not allow identification of separate lymphocytes as CD8^+^/FoxP3^+^.

### Ethical Consideration

This study was conducted in accordance with the guide-lines of the Declaration of Helsinki and its subsequent amendments, and informed consent was obtained from all patients.

### Statistical Analysis of the Results

Statistical analysis of the results was performed using Statistica v. 8.0 PL, Stat Soft Poland. The comparisons of the mean values of the analyzed parameters were performed using the nonparametric Mann–Whitney *U* test for the variables in the ordinal scale or the Student’s *t* test for variables in the interval scale. Normal distribution of the analysed values was verified using the Shapiro–Wilk test. The comparisons of DFS and OS of patients depending on the analysed factors were performed using the log-rank test whereas their graphic representation was prepared using the Kaplan–Meier method. The *p* = 0.05 level of significance was adopted for all statistical tests.

## Results

### The Results of the Evaluation of the Intensity and the Character of Macrophage Infiltration

Patients in whom recurrence was observed at the follow-up presented much more frequently with massive M2 macrophage infiltration CD68^+^/iNOS^−^ see assumptions described in “Material and Methods”. Their intensity within the connective tissue matrix of the tumor was classified in the overwhelming majority as moderate (2+) or intense (3+), with almost no infiltration intensity graded as (0) and (1+). These differences were highly statistically significant (*p* = 0.008).

Intense infiltration of M2 macrophages within the tumor stroma was related to shorter DFS (727 vs. 1397 days) and shorter OS (891 vs. 1411 days) with the levels of significance at *p* = 0.005 and *p* = 0.006, respectively. The relative risks of recurrence and cancer-related death were almost twice higher in the group of patients with intense infiltration of M2 macrophages within the tumor as compared to patients with no infiltration. The coefficients of the relative risks of recurrence and cancer-related death were RR 2.05, 95% CI 1.33–3.14, *p* = 0.001 and RR 2.08, 95% CI 1.28–3.39, *p* = 0.003, respectively.

**Fig. 2:**
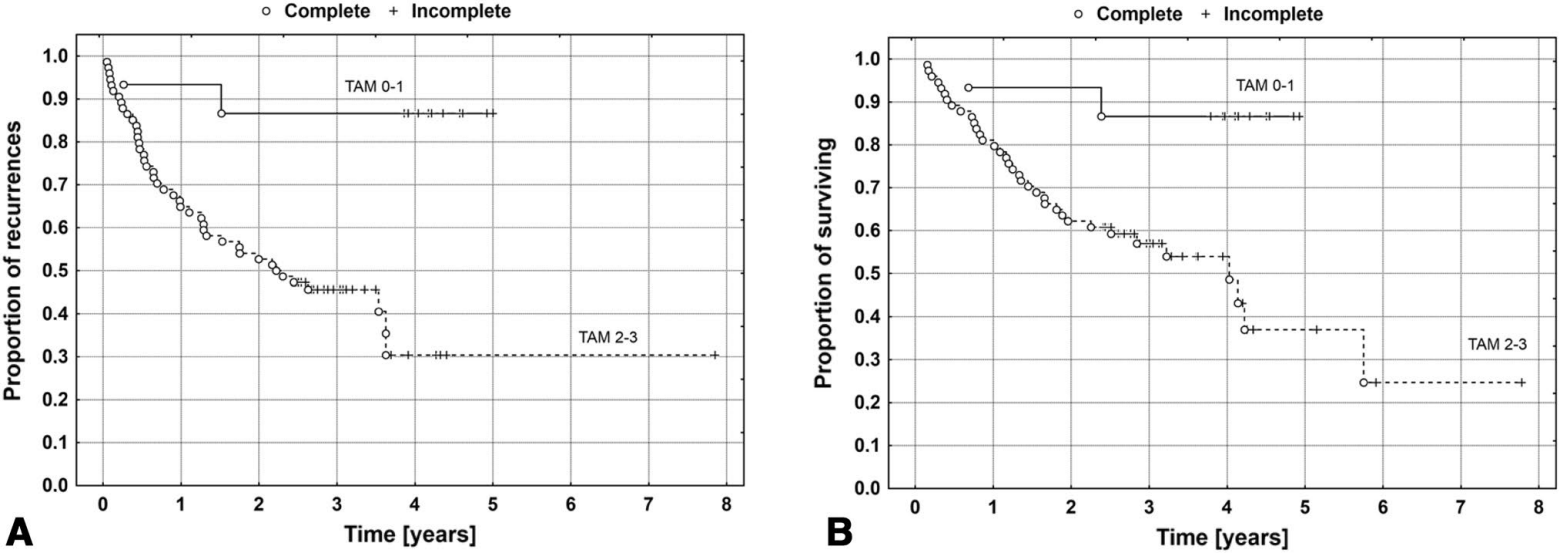
The Kaplan–Meier’s survival curves depending on the intensity of the infiltration of the tumor stroma by M2 macrophages for DFS (**a**) and OS (**b**). Sample size: TAM 0–1 *n* = 15; TAM 2–3 *n* = 74

**Fig. 3:**
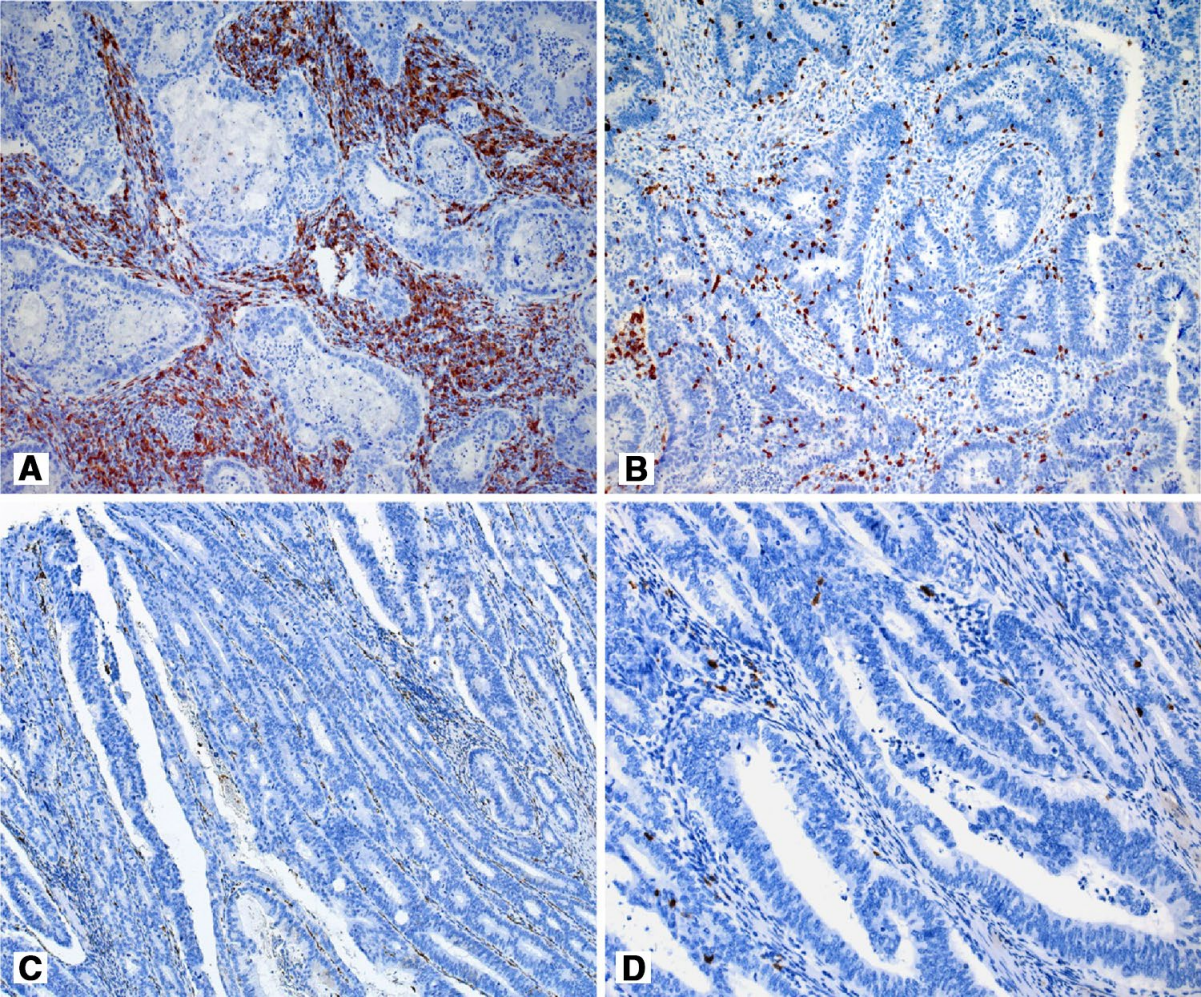
Infiltration of CD68^+^ macrophages (**a**) and numerous FoxP3^+^ lymphocytes (**b**) in the tumor stroma in a patient with the early systemic neoplastic process after surgery. Scant CD68^+^ and FoxP3^+^ infiltrations in patient with no symptoms of the disease at the time of the follow-up (**c** and **d**, respectively). Original magnification: ×200 (**a**–**c**); ×400 (**d**)

Survival curves for DFS and OS in the compared groups of patients are presented in Fig. 2, whereas representative examples of M2 macrophage infiltration of various intensity are presented in Fig. 3a, c.

The multifactorial analysis included three variables of potential prognostic value, i.e. the N feature of the TNM Classification of Malignant Tumours (TNM) classification, infiltration intensity of M2 macrophages and CD8^+^/FoxP3^+^ (Treg) lymphocytes within the tumor stroma. The analysis did not confirm the role of intensity of M2 infiltration as an independent prognostic factor both in relation to DFS and OS. However, a positive correlation of moderate intensity was confirmed between the intensity of infiltration of Treg lymphocytes and M2 within the tumor stroma (*R* = 0.54, *p* < 0.0001; Spearman rank correlation test).

In the case of M2 macrophage infiltration in the tumor front, an opposite tendency was observed, i.e. intense infiltration of TAMs at the tumor edge and the surrounding tissues was related to a lower recurrence rate. However, this relationship did not reach statistical significance (*p* = 0.061).

### The Results of the Assessment of the Number of Infiltrating T Lymphocytes

Details concerning intensity of immune cells infiltrates over particular clinico-histopathological categories are shown in Table 3.

**Table 3:** Descriptive statistics over clinico-histopathological categories

Feature	CD8^+^ (1/hpf)	FoxP3^+^ (1/hpf)	FoxP3/CD8 (%)	TAM
Age				
<median *n* = 43 (48.3%)	28.3 (14.0)	13.8 (11.9)	44.3 (25.0)	0:2; 1:6; 2:25; 3:10
≥median *n* = 46 (51.7%)	34.1 (19.6)	16.0 (12.5)	45.8 (24.8)	0:2; 1:5; 2:23; 3:16
Gender				
F: *n* = 44 (49.4%)	35.5 (19.1)* *p* = 0.03	16.5 (13.0)	43.4 (22.9)	0:2; 1:4; 2:22; 3:16
M: *n* = 45 (50.6%)	**27.4 (14.6)**	13.5 (11.3)	46.7 (26.6)	0:2; 1:8; 2:25; 3:10
Clinical stage at the time of surgery (T status)				
T1: *n* = 0 (0%)	–	–	–	–
T2: *n* = 2 (2.2%)	44.5 (21.9)	**33.5 (20.5)** ^+^ *p* = 0.03	72.6 (10.2)	0:0; 1:0; 2:1; 3:1
T3: *n* = 85 (95.6%)	30.6 (17.1)	**14.2 (11.7)**	44.0 (24.7)	0:4; 1:1l; 2:46; 3:24
T4: *n* = 2 (2.2%)	46.5 (16.3)	**27.5 (0.71)**	63.3 (23.7)	0:0; 1:0; 2:1; 3:1
Clinical stage at the time of surgery (N status)				
N0: *n* = 43 (48.3%)	**26.7 (14.1)** ^+^ *p* = 0.04	**10.8 (9.4)** ^+^ *p* = 0.0005	**38.9 (22.7)** ^+^ *p* = 0.001	0:3; 1:6; 2:25; 3:9
N1: *n* = 31 (34.8%)	**34.6 (19.8)**	**16.1 (12.7)**	**44.0 (24.3)**	0:1; 1:2; 2:16; 3:12
N2: *n* = 15 (16.9%)	**37.6 (17.6)**	**24.4 (13.0)**	**65.1 (22.5)**	0:0; 1:3; 2:7; 3:5
Clinical stage				
II: *n* = 43 (48.3%)	26.7 (14.1)* *p* = 0.02	10.8 (9.4)* *p* = 0.002	**38.9 (22.7)** ^*^ *p* = 0.02	0:3; 1:6; 2:25; 3:9
III: *n* = 46 (51.7%)	**35.6 (18.9)**	**18.8 (13.3)**	**50.9 (25.5)**	0:1; 1:5; 2:23; 3:17
Histological malignancy grading				
G1: *n* = 4 (4.4%)	40.5 (18.3)	14.3 (9.9)	38.3 (23.9)	0:0; 1:0; 2:3; 3:1
G2: *n* = 67 (75.3%)	29.1 (16.3)	14.1 (11.9)	44.8 (24.4)	0:3; 1:9; 2:38; 3:17
G3: *n* = 18 (20.3%)	37.5 (19.4)	18.3 (13.6)	47.7 (27.4)	0:1; 1:1; 2:7; 3:8

*P* statistical significance level of *t test and ^+^ANOVA test (significant differences marked with bold font); *CD8* ^*+*^ number of CD8-positive cells per high power field (hpf) of view, *FoxP3* ^*+*^ number of FoxP3-positive cells per hpf, *FoxP3/CD8* fraction of FoxP3-positive cells in the pool of CD8-positive cells, *TAM* intensity of CD68-positive cells infiltrates in the tumor tissue

The infiltration of T lymphocytes showing the expression of CD8^+^ surface antigens was observed both in the connective tissue of the tumor stroma and in malignant cells.

**Fig. 4:**
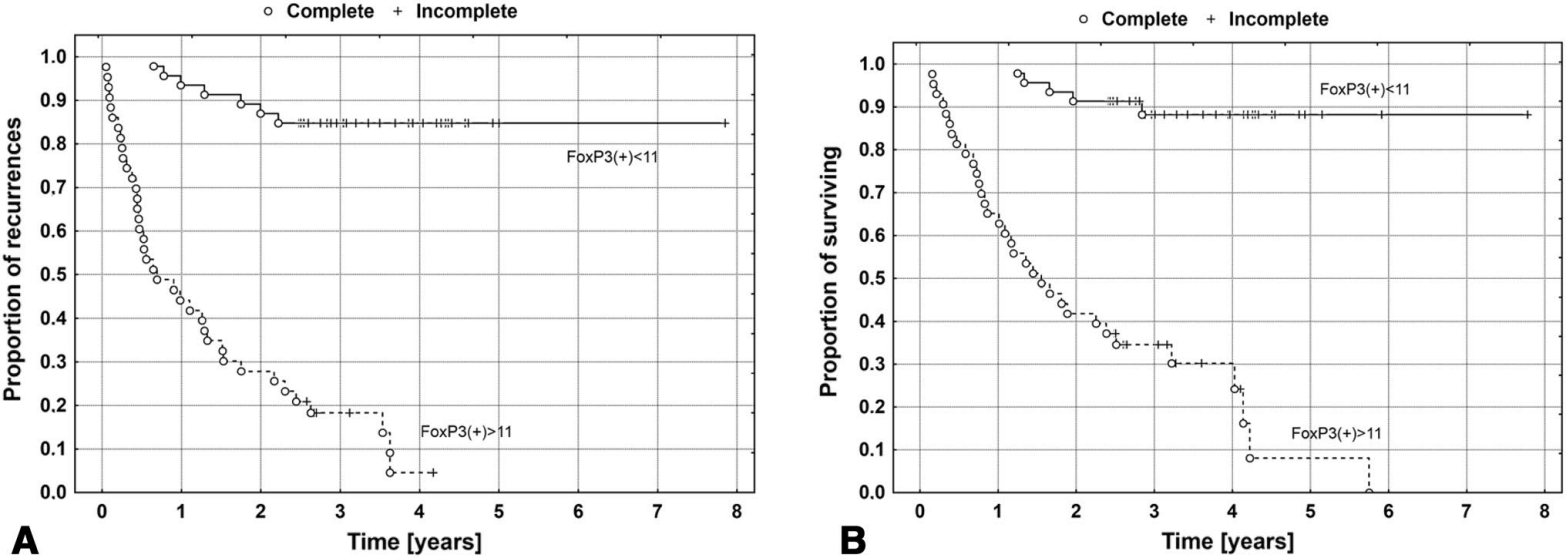
The Kaplan–Meier’s survival curves depending on the intensity of the infiltration of the tumor stroma by FoxP3^+^ Tregs for DFS (**a**) and OS (**b**)

In patients with recurrence at the follow-up, more intense CD8^+^ lymphocyte infiltration was observed with concurrent higher relative participation of CD8^+^/FoxP3^+^ (Treg) lymphocytes in the pool of CD8^+^, which was expressed by a higher number of Treg lymphocytes/hpf compared to the patients with no recurrence. These differences were highly statistically significant (*p* < 0.0001; see Tables 4, 5; Figs. 3a, d, 4).

**Table 4:** The summary of the results of tumor infiltration by lymphocytes and tumor associated macrophages in correlation to the occurrence of recurrences

Feature	Patients with recurrence *n* = 44	Patients with no recurrence * n* = 45	Level of statistical significance
The number of (cytotoxic) CD8^+^ T lymphocytes/hpf	Mean 38.4, SD 15.7	Mean 24.1, SD 15.9	*p* < 0.0001
The number of T regulatory FoxP3^+^/hpf	Mean 23.6, SD 11.3	Mean 6.1, SD 3.9	*p* < 0.0001
Participation of FoxP3^+^ lymphocytes in the pool of CD8^+^ lymphocytes (%)	Mean 61.6, SD 19.5	Mean 28.3, SD 17.2	*p* < 0.0001
Distribution of TAM infiltration intensities	0:4 (9.2%) 1:9 (20.4%) 2:22 (50.0%) 3:9 (20.4%)	0:0 (0.0%) 1:2 (4.4%) 2:26 (57.8%) 3:17 (37.8%)	*p* < 0.004

**Table 5:** The summary of the results of tumor infiltration by lymphocytes and tumor associated macrophages in correlation to the disease-related deaths

Feature	Patients died of disease, *n* = 51	Alive patients, *n* = 38	Level of statistical significance
The number of (cytotoxic) CD8^+^ T lymphocytes/hpf	Mean 39.1, SD 16.3	Mean 25.5, SD 15.7	*p* < 0.0002
The number of T regulatory FoxP3^+^/hpf	Mean 24.0, SD 10.9	Mean 8.1, SD 7.9	*p* < 0.0001
Participation of FoxP3^+^ lymphocytes in the pool of CD8^+^ lymphocytes (%)	Mean 62.9, SD 18.4	Mean 31.9, SD 20.3	*p* < 0.0001
Distribution of TAM infiltration intensities	0:4 (4.5%)	0:0 (0.0%)	*p* < 0.002
	1:9 (10.1%)	1:2 (2.2%)	
	2:27 (30.3%)	2:21 (23.6%)	
	3:11 (12.4%)	3:15 (16.9%)	

The presence of >11 FoxP3^+^ lymphocytes/hpf in the tumor stroma was related to an unfavorable prognosis regarding both DFS and OS (*p* < 0.0001, log-rank test). Intense FoxP3^+^ infiltration correlated with shorter DFS (463 vs. 1193 days) in the group with a lower number of FoxP3^+^ lymphocytes in the tumor stroma. Similarly, the mean time to death due to cancer in the group with the number of Treg lymphocytes above the median was 682 days as compared to 1256 days in the group with the number of Treg lymphocytes below the median.

Relative risk of recurrence in the group of patients with intense Treg infiltration was more than 12 times higher compared to patients with less intense infiltration (RR 12.3, 95% CI 5.44–27.9, *p* < 0.0001). Similarly, the risk of cancer-related death was significantly higher in this group (RR 12.5, 95% CI 4.9–32.4, *p* < 0.0001).

Next to the N feature, the number of Treg lymphocytes/hpf remained an independent prognostic factor in the Cox’s proportional hazards model which included three variables of a potential prognostic value, i.e. N feature of the TNM classification, intensity of M2 macrophages and FoxP3^+^ (Treg) lymphocytes. The results of the analysis based on the multifactorial model is presented in Table 6.

**Table 6:** The results of the Cox multifactorial risk model

Feature	RR	Standard error	Statistical significance, *p*	95% CI of RR
N	1.5	0.19	0.027	1.1–2.2
FoxP3^+^ <11	10.8	0.42	0.0001	4.7–24.7

*RR* relative risk, *95% CI* 95% confidence interval

## Discussion

During carcinogenesis, TAMs change their phenotype and the manner of interaction with the surroundings, i.e. stromal cells, lymphocytes and malignant cells. TAMs, which in time polarize toward M2 phenotype with poor antigen-presenting capability and immunosuppressive activity by releasing immunosuppressive factors (IL-10, TGF-β, EGF, VEGF, MMPs), are regarded to be pro-tumor for many cells (Kryczek et al. 2006). However, despite the fact that M2 TAMs play adverse role related to a poorer prognosis for the majority of tumors (Chai et al. 2008; Kryczek et al. 2006; Tsutsui et al. 2005), in the case of CRC it is not so evident. Some authors report that high density of TAMs is associated with an unfavorable prognosis (Cui et al. 2013; Tan et al. 2005), whereas other reports demonstrate contrary results (Algars et al. 2012; Gulubova et al. 2013; Forssell et al. 2007; Zhou et al. 2010). The results indicating a favorable prognosis related to an increase in the density of M2 TAMs were obtained from the tumor front and not the tumor itself. It is stressed that an improvement in survival is related to high infiltration by macrophages at the tumor front as an expression of a strong immune defense reaction, particularly at the early stages of carcinogenesis. Later an increase in the density of M2 TAMs in necrotic tumor tissues was observed with the concurrent increase in M1 TAMs (Forssell et al. 2007; Shih et al. 2006; Tan et al. 2005). The ambiguity of the results may be caused by a “variable involvement” of TAMs in various locations at various stages of carcinogenesis or a different distribution of M2 TAM subpopulation which is assayed using four different methods (Shih et al. 2006). Only the outcome of TAM activities results in either pro- or anti-tumor influence on inflammation-dependent tumor, i.e. CRC. It should be considered that tumor cells are able not only to block the activity of TAMs in the tumor, but also to modify the activity of TAMs to promote survival and progression of the tumor (Shih et al. 2006). It seems that within the tumor front this modulating influence of tumor cells is the lowest and the character of TAM activity in this area is anti-tumor. Within the tumor, especially in more advanced stages, the outcome of the microenvironment activity on TAMs is pro-tumor.

In our study, in CRC patients with stages IIA, IIIB, and IIIC, a high density of iNOS^−^ TAMs in the tumor itself is related to shorter survival time and a high risk of recurrence, which is consistent with the reports of Cui et al. (2013) and Tan et al. (2005). However, it significantly correlates with the number of Treg lymphocytes within the tumor stroma and thus it does not constitute an independent prognostic factor. Furthermore, this relationship may be an indirect evidence of the TAM role in programming the immunomodulatory function of FoxP3^+^ lymphocytes within the tumor stroma, which is also reported by other authors (Gabrilovich et al. 2012; Galon et al. 2006; Ramanathan et al. 2008). However, an opposite tendency was observed within the tumor front, i.e. an increase in the intensity of iNOS^−^ TAMs infiltration in the tumor front was related to a better prognosis. This relationship, however, did not reach statistical significance (*p* = 0.061).

In the present study, the method of an indirect assessment of M2 macrophage infiltration by subtracting the number of M1 iNOS^+^ macrophages from the pool of all CD68^+^ macrophages infiltrating the tumor was intended to identify the M2 subtype with consideration given to a number of subtypes characterized by diverse expression profiles of specific markers. The lack of the possibility to confirm a simultaneous expression of markers in specific single cells in the case of CD68^+^/iNOS^−^ as well as CD^+^/FoxP3^+^ phenotypes constitutes a limitation of the adopted methodology. However, it could be overcome by the evaluation of immunohistochemistry reactions in the corresponding areas on the serial histological sections. It seems that this simplification does not affect the prognostic value for such determination of M2 TAMs and Treg lymphocytes. Certainly, the use of combination of single-specific markers for M2 TAMs in the same section would eliminate the omission of a small number of macrophages in this subpopulation but it would render the in situ determination technically impossible and it would increase the costs and, consequently, the availability of the test. We are of the opinion that not covering of all M2 phenotypes should not have a significant influence on its practical usefulness and the intensity of CD68^+^/iNOS^−^ macrophage infiltration within the tumor stroma seems to be a good prognostic factor in CRC.

The FoxP3^+^ transcription factor, which plays a significant role in the regulation of the development and the activity of the immune system, is treated as an immunosuppressive factor. It is considered the most specific marker for CD4^+^/CD25^+^ and CD8^+^/CD25^+^ Treg lymphocytes, although its expression was also confirmed in tumor cells (Chaput et al. 2009; Takenaka et al. 2013). The majority of studies investigate the subpopulation of Treg cells showing the expression of CD4^+^/CD25^+^ and the knowledge related to them is broader. A high density of FoxP3^+^ Treg cells infiltrating the tumor stroma is considered a poor prognostic factor in numerous types of tumor (Gao et al. 2007; Hiraoka et al. 2006; Kobayashi et al. 2007; Merlo et al. 2009; Sasada et al. 2003; Xue et al. 2010). However, the study results on CRC are ambiguous (Ling et al. 2007; Loddenkemper et al. 2006; Salama et al. 2009). Some authors recognize FoxP3+ Tregs as an unfavorable prognostic factor (Grimmig et al. 2013; Michel et al. 2008), whereas others confirm that a high density of Tregs is related to a good prognosis (Ladoire et al. 2011). Another group of studies negates its value (Chaput et al. 2009; Loddenkemper et al. 2006), whereas Kim et al. (2013) are of the opinion that it is high expression of FoxP3^+^ in tumor cells and not in Treg lymphocytes that is related to a bad prognosis as compared to patients with low FoxP3^+^ expression.

The results of our study on CD8^+^/FoxP3^+^ lymphocytes indicate that the increase in the intensity of the infiltration of the tumor stroma by such defined subpopulation of Treg cells may be considered an unfavorable and independent prognostic factor. This may be attributable to their suppressive influence on anti-tumor activity of the immune system and may be the result of a subtle regulation occurring between lymphocytes related to CD8^+^/FoxP3^+^ tumor and macrophages related to CD68^+^/iNOS^−^ tumor whose co-location in the tumor stroma was confirmed in the present study.

As in the case of macrophage determination, it should be considered that the applied single staining method of the corresponding areas in serial sections renders the identification of individual lymphocytes as CD8^+^/FoxP3^+^ impossible and it merely allows an overall assessment of these areas from a statistical perspective.

In conclusion, our retrospective study comprising 89 CRC patients with stages IIA, IIIB, and IIIC confirms that infiltration of the tumor stroma by CD68^+^/iNOS^−^ TAMs and CD8^+^/FoxP3^+^ Tregs is a negative prognostic factor and there is a positive correlation between these types of infiltration.

The intensity of the infiltration of the tumor stroma by CD8^+^/FoxP3^+^ lymphocytes may constitute an independent prognostic factor in the group of CRS patients with stages IIA, IIIB, and IIIC, which requires confirmation in prospective studies.

The proposed methodology of TAM and Treg phenotype identification in histological sections is mainly directed at practical use in routine clinical management.
